# Enhanced Near-Infrared Ultra-Narrow Absorber Based on a Dielectric Nano-Resonant Ring for Refractive Index Sensing

**DOI:** 10.3390/s23208402

**Published:** 2023-10-12

**Authors:** Xingyu Li, Dingquan Liu, Junli Su, Leihao Sun, Haihan Luo, Gang Chen, Chong Ma, Qiuyu Zhang

**Affiliations:** 1 Shanghai Institute of Technical Physics, Chinese Academy of Sciences, Shanghai 200083, China; lixy12@shanghaitech.edu.cn (X.L.); sujl@shanghaitech.edu.cn (J.S.); lhsun23@m.fudan.edu.cn (L.S.); gangchen@mail.sitp.ac.cn (G.C.); machong@mail.sitp.ac.cn (C.M.); zhangqy@shanghaitech.edu.cn (Q.Z.); 2 School of Physical Science and Technology, ShanghaiTech University, Shanghai 200031, China; 3 School of Optoelectronics, University of Chinese Academy of Sciences, Beijing 100049, China

**Keywords:** narrow-band absorption, ITO, near-field enhancement, refractive index sensing

## Abstract

In this paper, a plasmon resonance-enhanced narrow-band absorber based on the nano-resonant ring array of transparent conductive oxides (TCOs) is proposed and verified numerically. Due to the unique properties of TCOs, the structure achieves an ultra-narrowband perfect absorption by exhibiting a near-field enhancement effect. Consequently, we achieve a peak absorption rate of 99.94% at 792.2 nm. The simulation results indicate that the Full Width Half Maximum (FWHM) can be limited to within 8.8 nm. As a refractive index sensor, the device reaches a sensitivity S of 300 nm/RIU and a Figure of Merit (FOM) value of 34.1 1/RIU. By analyzing the distribution characteristics of the electromagnetic field at the 792.2 nm, we find high absorption with a narrow FWHM of the ITO nano-resonant ring (INRR) owing to plasmon resonance excited by the free carriers at the interface between the metal and the interior of the ITO. Additionally, the device exhibits polarization independence and maintains absorption rates above 90% even when the incident formed by the axis perpendicular to the film is greater than 13°. This study opens a new prospective channel for research into TCOs, which will increase the potential of compact photoelectric devices, such as optical sensing, narrowband filtering, non-radiative data transmission and biomolecular manipulation.

## 1. Introduction

In recent years, metasurfaces have garnered significant attention in the fields of optics and communication due to their ability to manipulate the phase, amplitude, and polarization of incident electromagnetic waves through various frequency ranges [1,2,3,4]. One of the most important applications of metasurfaces lies in their ability to be utilized as absorbers. Metasurface absorbers have attracted considerable interest due to their vast potential applications in energy, detection, and sensing areas [5,6]. After years of extensive research, various types of metasurface absorbers have been designed and developed, including broadband absorbers [7,8], narrowband absorbers [9], and tunable absorbers [10]. The narrowband absorbers are particularly intriguing due to their superior efficiency in comparison to wideband absorbers when utilized in optical sensors and thermal transmitters. Moreover, within the area of optical detection, broadband absorbers fail to meet application requirements, while narrowband absorbers exhibit higher quality factors (FOM) and sensitivity (S) [11]. Thus, narrowband absorbers are extensively employed for effective sensing in the contexts of biological materials [12], viruses [13], temperature [14], humidity [15], and gas [16].

Various structures of narrowband absorbers have been proposed, including split-ring resonator arrays, metal–dielectric periodic gratings, metal–dielectric–metal and full-metal periodic configurations [17,18,19,20,21]. Meng et al. proposed using Ag grating and air medium-based surface plasmon resonance to achieve narrow-bandwidth absorption at 1400 nm with an FWHM of only 0.4 nm [17]. Liu et al. utilized a split-ring metasurface to design a refractive index sensor with an impressive FWHM of 8 nm and FOM of 56.5 1/RIU [19]. Fu et al. achieved exceptional absorption performance by preparing an Ag array absorber, which could reach 99.2% absorption with a remarkable FWHM of 24 nm [20]. Kang S et al. fabricated a cross-shaped nanoarray consisting of Au on a Si substrate, and deposited a thin layer of Au on the opposite side of the Si to impede transmission [21]. Experimental results have demonstrated that this absorber exhibited narrow-bandwidth absorption in the terahertz band. However, the inherent ohmic loss of metals, which are intensively applied in narrowband sensors, inhibits the resonance of plasmon due to joule and radiation losses. These losses can not only result in a widened spectrum of device characteristics, but also weakened response strength. Therefore, some researchers anticipate employing materials with minimal or negligible loss as alternatives for plasmon metals, such as Si [22], Ge [23], GaAs [24] and other semiconductor materials. In particular, heavily doped complementary metal oxide with low loss (i.e., TCOs) provides an additional option for plasmon resonance materials. These TCOs are degenerate semiconductors with carrier concentrations ranging from 10^20^ to 10^21^ cm^−3^. In general, TCOs mainly consist of Sn-doped indium oxides, Al- or Ga-doped Zinc oxides, and mixed families of these oxides. Indium tin oxide (ITO), as the most commonly utilized and relatively most mature commercial TCO film material, still holds great potential for use in scientific research. Li et al. demonstrated that by finely synthesizing ITO nuclear magnetic resonance through a random array of ITO nanorods, the intrinsic plasmon frequency can be altered to achieve surface plasmon infrared enhanced absorption [25]. In addition, Min et al. employed an ITO film to construct a multi-spectral integrated stealth device that exhibits low infrared emissivity and wideband microwave absorption, while simultaneously ensuring maximum visible light transparency in accordance with the engineering requirements of a multi-spectral integrated compatible design [26]. The viability of this design has been demonstrated. However, the above achievements have only focused on metasurfaces with broadband absorption. There are few studies exploiting ITO materials to achieve ultra-narrowband absorption in the near-infrared range.

In this study, we deposited an ITO film onto a Si substrate and calculated the optical parameters of the ITO film across the visible to near-infrared spectrum via ellipsometry. Subsequently, we theoretically designed a narrowband absorber consisting of an INRR with a simple structure. By analyzing the distribution characteristics of the electromagnetic field at 792.2 nm, we found high absorption with a narrow FWHM produced by the INRR owing to plasmon resonance excited by the free carriers at the interface between the metal and the interior of the ITO. Besides this, compared with the same metasurface formed by Au, INRR shows a narrower FWHM and a greater performance in sensing, which is attributed to the bulk electron distribution in ITO rather than the surface distribution on Au. Furthermore, by adjusting the structural parameters of the INRR, we manipulated the resonance position and achieved varying degrees of absorption across different spectra. Ultimately, the sensing capabilities of the sensor S and FOM are 300 nm/RIU and 34.1 1/RIU, respectively. The device maintains absorption rates above 90% even when the incident formed by the axis perpendicular to the film is greater than 13°, and represents polarization independence due to its high geometric symmetry. These results demonstrate the great potential of using our designed structure in optical devices.

## 2. Experiment and Design

ITO films with a thickness of 100 nm were prepared on a Si substrate by radio frequency (RF) magnetron sputtering. Subsequently, the ITO films were measured with a spectral range of 350–1500 nm per 2 nm at three incident angles of 65°, 70° and 75° using a spectral ellipsoid (SE) instrument (V-VASE, J.A. Woollam, Lincoln, NE, USA). Obtaining ψ and δ data from multiple incident angles at each measured wavelength can significantly enhance the accuracy of fitting results, rendering these an invaluable asset in determining one or more unique optical model.

The optical coefficient of ITO film in the visible range is determined by the Cauchy model, taking into account its transparency as a medium within this range [27]. In the near-infrared range, it is commonly expected that the free carrier conductivity of the film can be determined by fitting the material’s absorption. The observed absorption is attributed to the presence of free charge carriers (Drude edges) within the film. The refractive index and absorption coefficient are modeled using one or more Lorentz harmonics, based on the Drude model [28]. For our plated ITO film, we utilized two Drude oscillators and two Lorentz (harmonic) oscillators to model the refractive index of the ITO film in the infrared region. The mathematical model for a collection of Lorentz oscillators is:

(1) $$\tilde{\varepsilon }(hv)=\varepsilon_{1}+i\varepsilon_{2}=\varepsilon_{1\infty }+\sum_{j}\frac{A_{j}}{\left(E_{j}^{2}-{\left(hv\right)}^{2}-iB_{j}hv\right)}$$

**Figure 1:**
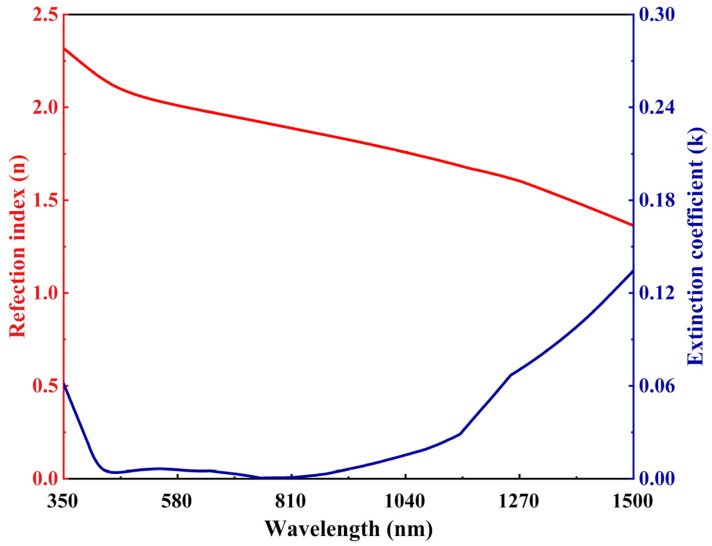
Fitting values of optical constants of ITO.

For the *j*th oscillator, *A_k_* is the amplitude, *E_k_* is the center energy, *B_k_* is the broadening of each oscillator, hv is the photon energy in eV, and $\varepsilon_{1\infty }$ is an additional offset term defined in the model. By combining the mentioned two-stage fitting calculations from visible to near-infrared regions, Figure 1 illustrates the results of the ITO optical parameters, including n and k values, across a broad spectral range from 350 to 1500 nm. The optical parameters of Au are described by the Johnson model [29].

**Figure 2:**
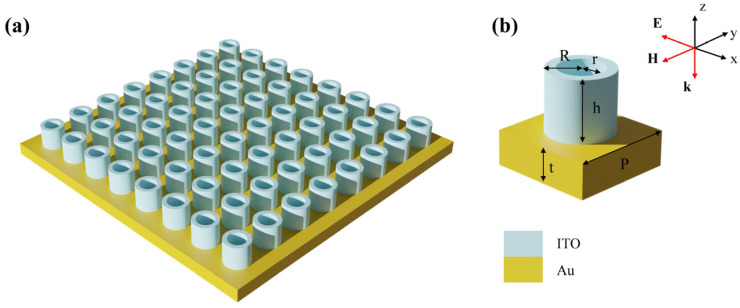
Schematic diagram of the absorber array. (**a**) Schematic diagram of array structure. (**b**) A single-cell structure and its related structural dimensions.

The INRR was designed and calculated using FDTD Solutions from the LUMERICAL Corporation based on the data obtained from SE. The designed metasurface absorber is composed solely of INRR sitting on a fold substrate, as depicted in Figure 2a. Figure 2b shows the schematic diagram of a unit cell and its corresponding electromagnetic field direction. Herein, *R*, *r*, and *h* represent the outer radius, inner radius, and height of the INRR, respectively. The thickness of the Au layer and the period of the structure are denoted by *t* and *P*, respectively. The structural parameters are R=190 nm, r=130 nm, h=350 nm, and P=600 nm. Taking into account the influence of the metal skin’s depth, a thickness of t=200 nm is chosen for the bottom reflecting film made of Au to ensure the complete reflection of incident electromagnetic light waves. In the fitting model, incident electromagnetic light waves propagate in the negative direction along the z direction and are polarized in the x direction (i.e., transverse electric wave). The x and y directions are subject to periodic boundary conditions, while the z direction is equipped with a perfect matching layer (PML). To ensure accuracy, a 2 nm grid coverage is employed in all directions surrounding the INRR.

For future manufacturing applications, we outline the following preparation process. The top INRR can be fabricated through a combination of metal evaporation, RF magnetron sputtering and electron beam lithography (EBL) during the device preparation process [30]. In detail, the Au reflective layer is first deposited onto the substrate through electron beam evaporation, followed by a coating of photoresistor on its surface. Upon exposure and development, the pattern from the mask plate is transferred to the photoresistor to form regions with distinct properties. The ITO film layer is subsequently deposited on the entire region via magnetron sputtering. A negative resist film is spun onto the sample by EBL, which directly draws a ring pattern with nanoscale characteristic dimensions, and we then induce coupled plasmon etching. Finally, the residual impedance is eliminated through the glass process, and the metasurface absorber displayed in Figure 2 can be constructed.

## 3. Results and Discussion

**Figure 3:**
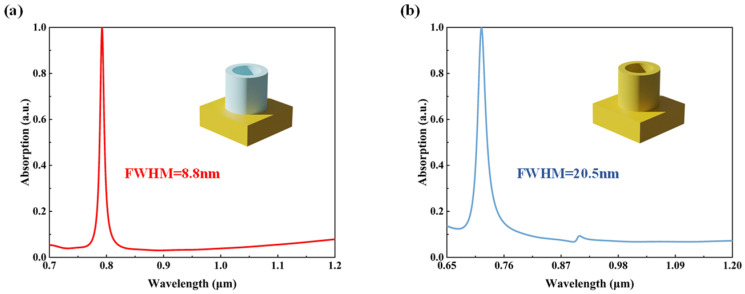
Absorption spectra and FWHM of nano-resonant rings. (**a**) INRR structure; (**b**) ANRR structure.

The INRR absorption spectrum is shown in Figure 3a, where the sharp resonance peak of complete absorption (|A|=1) occurs at a near-infrared wavelength of 792.2 nm, and the FWHM is only approximately 8.8 nm. In general, perfect absorption can also be achieved in the resonance mode based on a Au nano-resonance ring (ANRR). However, due to the inherent ohmic loss suffered by the metal, it is challenging for the ANNR to further compress the spectrum into a narrower FWHM. The INRR can effectively reduce dissipation loss and enable the perfect absorption of narrow line widths. To provide a comparison, we also calculated the absorption spectra of the ANNR, as depicted in Figure 3b. Compared with the INRR, this structure only alters the material of its top dielectric layer. It is evident that the ANRR exhibit a near-perfect absorption peak at 717.7 nm with an FWHM of 20.5 nm, which reveals the pivotal role played by the top INRR in achieving high and narrowband absorption in near-infrared wavelengths.

### 3.1. Electromagnetic Field Analysis

**Figure 4:**
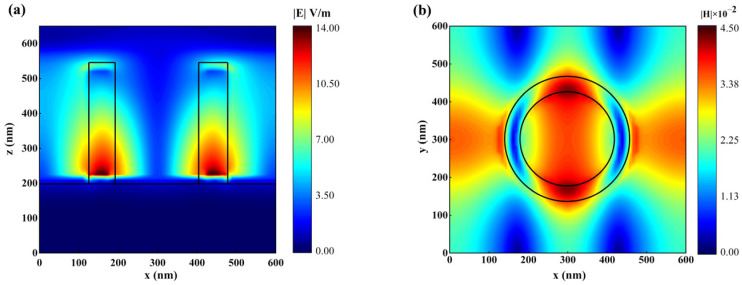
Electric and magnetic fields of INRR. (**a**) The x–z axis electric field; (**b**) magnetic field of the x–y axis.

To unveil the underlying mechanism of absorption, we investigated the electromagnetic field response of the nanostructure at 792.2 nm. Figure 4a,b show the electric field along the x–z direction and the magnetic field along the x–y direction of the INRR, respectively. Notably, the intense electric field in the INRR is primarily localized at both ends of its diameter along the x direction, and is proximal to the interface between Au and ITO materials. The magnetic field induced by the strong electric field envelops and distributes perpendicularly in the direction of the electric field. Specifically, incident light stimulates non-uniformly localized electric fields along the x direction of the resonant ring, resulting in dissimilar charges being distributed on both sides of the inner diameter of the ring along this axis. Meanwhile, opposite charges are distributed on both inner and outer sides of the ring. A significant number of opposite charges are also present on the outer surfaces of different rings among the neighboring cells. The distribution of electric field is equivalent to the primary excitation of three pairs of electric dipoles within a cell, as well as an electric dipole between neighboring cells. These electric dipoles will further stimulate the magnetic field perpendicular to the plane of incidence for light. Therefore, the incident electromagnetic wave excites localized surface plasmon resonance (LSPR) in INRR. The response of free carriers to light at a specific geometric size is known as the LSPR effect. In the nanostructure, the LSPR is primarily composed of free electrons on the surface of the Au layer. Additionally, the ITO material promotes absorption by providing numerous free charges due to its high carrier concentration exceeding 4.509 × 10^20^ cm^−3^ [31]. Therefore, the device exhibits an infrared near-field enhanced absorption effect, resulting in the perfect absorption of near-infrared light with a wavelength of 792.2 nm. Due to the lower loss of the dielectric material, only the electromagnetic field close to the central wavelength can be localized here, leading to a relatively narrow FWHM.

**Figure 5:**
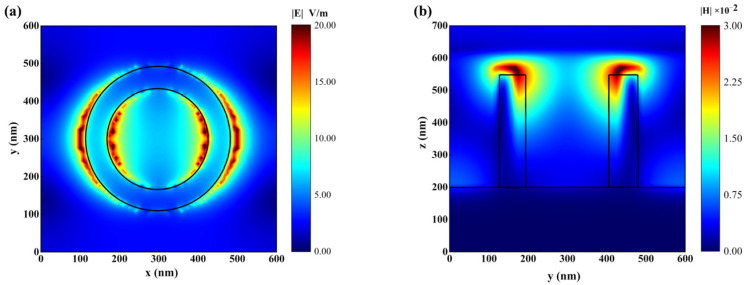
Electric and magnetic fields of ANRR. (**a**) Electric field along the x–y axis; (**b**) y–z axis magnetic field.

To more intuitively compare the essential differences between INRR and ANRR, we analyzed the corresponding electric and magnetic field distributions of the latter at resonant wavelengths. As illustrated in Figure 5, the electric field intensity is confined to both termini of the outer and inner rings of the Au nano-ring, while the magnetic field is predominantly localized on the upper surface of the ANRR. The phenomenon typifies the LSPR effect and manifests enhanced absorption performance [32]. The energy is restricted and an absorption peak is generated due to the localized surface electronic polarization. The non-uniform surface distribution of the electric field on ANRR leads to high-order LSPR absorption modes. In the INRR, as a dielectric material, the carrier distribution of ITO can be dispersed within the ring structure. The distinctive phenomenon of absorption spectrum expansion is reduced because of the reduction in high-order plasmon absorption resulting from the uneven charge distribution. Thus, the FWHM of the INRR is less than half that of the ANRR, while generating a resonant magnetic field intensity 1.5 times greater. The contrastive analysis indicates that the metasurface of the INRR leads to a greater field enhancement and application potential of the ultra-narrowband perfect absorption.

### 3.2. Geometric Parameter and Fabrication Tolerances

The absorption efficiency and position of the absorption peak of a metasurface absorber are generally determined by the geometric structure parameters. To gain the best absorption performance of the INRR, it is necessary to consider changes in the geometric parameters of the resonant ring [33,34,35]. In the case of the standard parameters R=190 nm, r=130 nm, h=350 nm, and P=600 nm, we independently analyzed the individual geometry parameters while maintaining the rest of the parameters as constants.

After parametric scanning, we determined that a slight variation in the period of the INRR has negligible impact on absorption performance. Specifically, within a period range of 550–800 nm, only a redshift in resonance position occurs while maintaining high absorption efficiency. The INRR we have designed will exhibit a circumferential electric field at the resonance response wavelength, as previously mentioned. When the distance between the two rings does not change too much (between 250 and 350 nm), the resonance wavelength will only transform due to the contraction or elongation of the distance, yet without significantly affecting the resonance intensity. At the resonance position of the absorption peak, we will study the impacts of thye INRR’s height, outer radius, inner radius and period, respectively, on its absorption performance.

**Figure 6:**
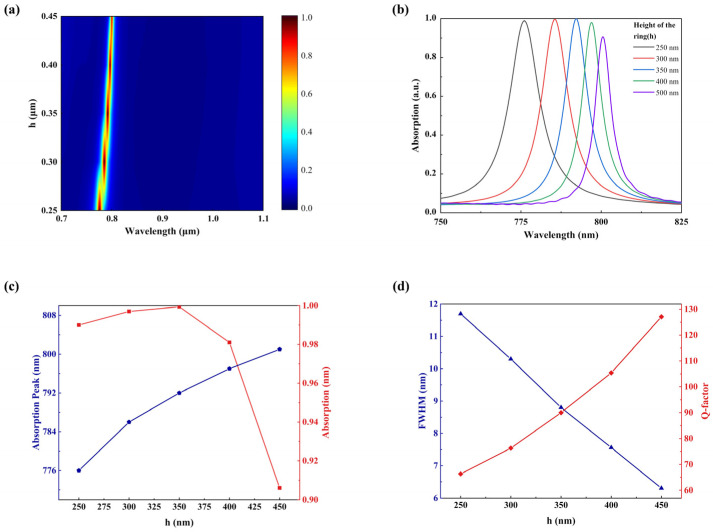
Dependence of absorption characteristics of INRR on the value of *h*. (**a**) Absorption mapping with different heights. (**b**) The simulated absorption spectrum from 250 to 450 nm with height *h*. (**c**) Resonance wavelength (blue line) and absorption intensity (red line) corresponding to different heights *h*. (**d**) The corresponding FWHM (blue line) and Q-factor (red line) as a function of the height *h*.

Figure 6 shows the impact of the INRR’s height on absorption efficiency. Figure 6a maps the impact of *h* on wavelength and absorption value, and Figure 6b shows the absorption spectrum of individual *h*. As illustrated, an increase in *h* results in a redshift of the resonance wavelength, followed by a decline in absorption rate after reaching its peak at h=350 nm. A nearly linear redshift of the absorption peak is observed in Figure 6c, while maintaining high absorption rates (>90%). Furthermore, Figure 6d presents the calculation of FWHM and Q-factors for absorption peaks at various *h* values. The performance factor Q of sensors is defined as Q=λ/FWHM [36,37,38], as represented by the blue line in Figure 6d. It can be noted that as the height increases, the optical path of light traveling through the material gradually increases, resulting in a more challenging excitation with a higher resonance intensity. However, the FWHM and Q-factor exhibit the opposite trend, while the narrowest FWHM is observed at h=450 nm, where an absorption intensity of only 90.5% is achieved. The competitive cooperation of FWHM and Q-factor implies that the absorption intensity cannot be overlooked in pursuit of a larger Q-factor, as it would compromise the perfect absorption characteristics of the structure.

**Figure 7:**
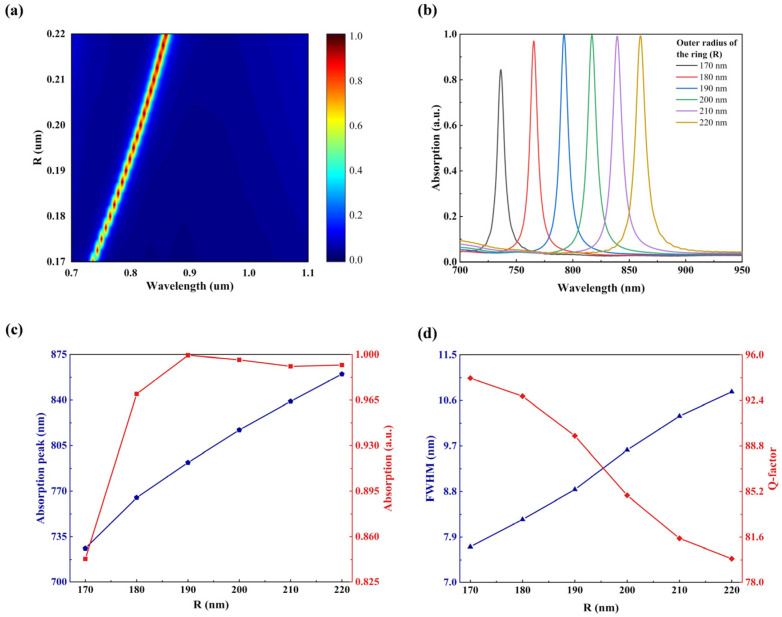
Dependence of absorption characteristics of INRR on outer radius *R*. (**a**) Absorption mapping with different *R*. (**b**) The simulated the absorption spectrum of *R* from 0.10 to 0.15 um. (**c**) Resonance wavelength (blue line) and absorption intensity (red line) corresponding to *R*. (**d**) The corresponding FWHM (blue line) and Q-factor (red line) as functions of the *R*.

Subsequently, we proceeded to adjust the outer radius *R* of the INRR. As shown in Figure 7, with the increase in the ITO nano-resonant ring *R*, the filling ratio of the resonant ring on its surface gradually increases, resulting in great diversity between the inner and outer radii. Fundamentally, an increase in *R* leads to an increased interaction distance between different charges, which induces a smaller recovery coefficient (RC) for oscillating electrons in response. Additionally, relaxation effects caused by the nonuniform distribution of electric fields near the nanostructure result in redshift phenomena in the resonance peaks. It can be seen in Figure 7c that *R* exhibits a sharp increase beyond 170 nm. However, the absorption intensity begins to decline after reaching its peak at 190 nm. As shown in Figure 7d, the calculation results indicate that as the *R* is raised, there is a corresponding increase in FWHM and a decrease in the Q-factor, which demonstrates an inverse relationship.

**Figure 8:**
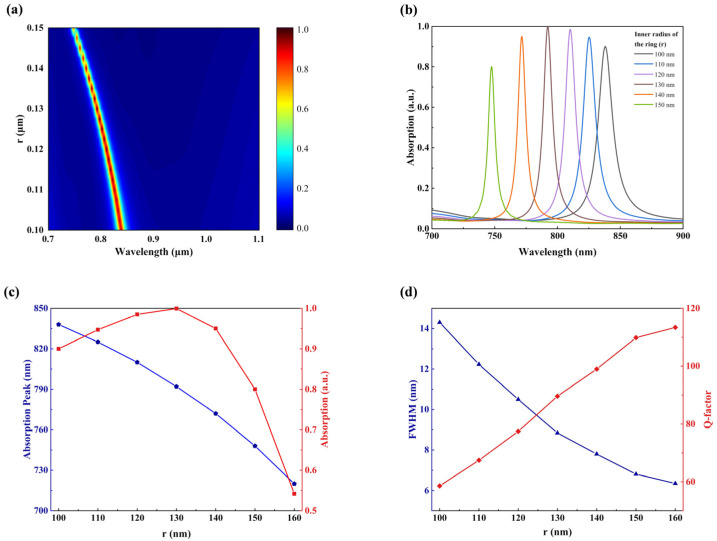
Dependence of absorption characteristics of INRR on inner radius *r*. (**a**) Absorption mapping with different *r*. (**b**) The simulated absorption spectrum of *r* from 100 to 150 nm. (**c**) The resonance wavelength (blue line) and absorption intensity (red line) corresponding to *r*. (**d**) The corresponding FWHM (blue line) and Q-factor (red line) as functions of *r*.

Furthermore, we tuned the inner radius r of the ITO resonant ring. Figure 8a,b demonstrate the influence of r on both the wavelength and magnitude of the absorption peak. As r increases, the filling ratio of the ITO resonant ring decreases. Similar to the influence mechanism of *R* mentioned above, as r increases from 100 nm, the absorption peak gradually shifts towards shorter wavelengths (blue shift), while the absorption value increases gradually. However, once r exceeds 130 nm, a sharp decline in absorption rate can be seen. Furthermore, Figure 8c,d demonstrate fluctuations in resonance wavelength, Q-factor, and FWHM as a function of r. As depicted in Figure 8d, an increase in the value of r leads to a reduction in FWHM and an enhancement in the Q-factor, which means the two parameters exhibit opposite trends. Nevertheless, it should be noted that both the absorption rate and resonance wavelength of the nano-resonant ring are significantly influenced by its inner radius r.

**Figure 9:**
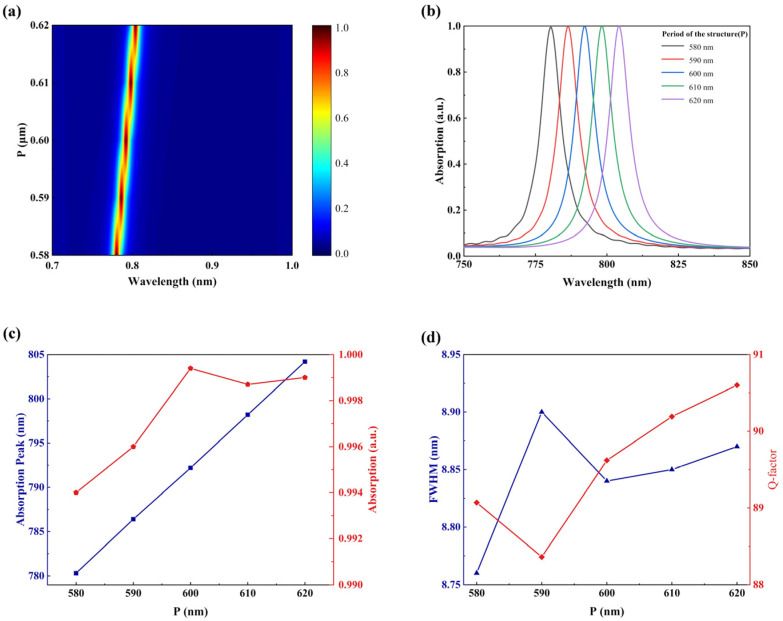
Dependence of absorption characteristics of INRR on period *P*. (**a**) Absorption mapping with different *P*. (**b**) The simulated absorption spectrum of *P* from 580 to 620 nm. (**c**) The resonance wavelength (blue line) and absorption intensity (red line) corresponding to *P*. (**d**) The corresponding FWHM (blue line) and Q-factor (red line) as functions of *P*.

To account for potential manufacturing errors in the entire device, we have evaluated the impact of variable period P on absorption efficiency. We can see from the results presented in Figure 9 that there is no significant alteration in the absorption rate when the value of P varies within a range of nanometers. The phenomenon arises due to the shortening or extension of the distance between resonant rings, which leads to a redshift or blueshift in the resonance wavelength without significantly impacting strength. The results will also hold guiding significance in the determination of structural parameters. Based on the usage requirements of refractive index sensors, we have selected parameter P=600 nm as one of the geometric parameters due to its high absorption rate and the fact that it has the narrowest FWHM with high absorption performance.

### 3.3. Sensing Characteristics of INRR and ANRR

**Figure 10:**
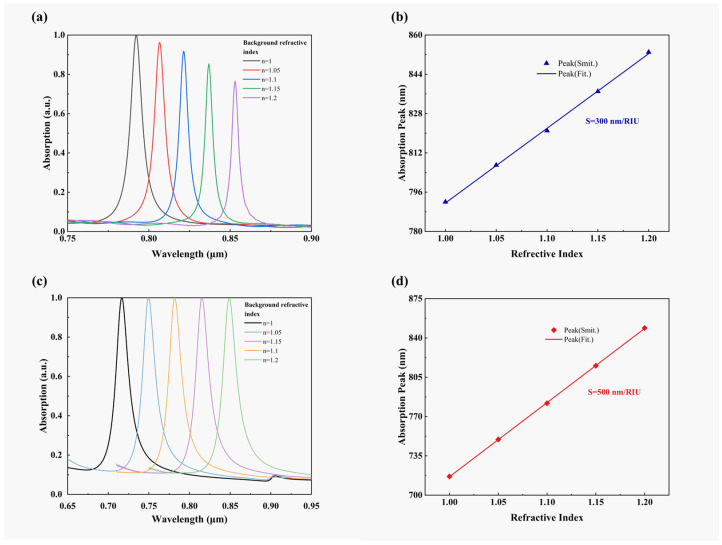
Refractive index changes from 1 to 1.2 with a step size of 0.05. (**a**) The absorption rate of NRR varies with refractive index. (**b**) The position and linear fitting of the resonant wavelength of INRR. (**c**) A two-dimensional diagram of the absorptivity of ANRR as a function of refractive index. (**d**) Resonance wavelength position and linear fitting of ANRR.

When the geometric structure of the device remains at the values chosen and the refractive index n of the surrounding environment increases, the equivalent electric dipole moment in the excited plasmon also increases correspondingly. Compared with the distance between hetero charges when n=1, when n increases, the equivalent distance increases and the recovery coefficient decreases, so the response wavelength will be red-shifted. The characteristic effect whereby the resonant wavelength shifts with variations in ambient refractive index is extensively applied in refractive index sensors [39,40,41]. Based on the exceptional ultra-narrowband perfect absorption properties of the INRR, we aim to further investigate its potential for use in sensor detection applications. In this study, we conducted simulations to investigate the correlation between the absorption peak position of ITO nanostructures and changes in their surrounding environment, specifically examining variations in refractive index within the range of n=1~1.2. Two-dimensional absorption spectra images under varying refractive indexes ranging from 1 to 1.2 with a step size of 0.05 are depicted in Figure 10a, where the resonance wavelength undergoes redshift as the refractive index increases. Subsequently, the resonant wavelength is analyzed and graphed as a function of refractive index, depicted in Figure 10b. The data points represent the corresponding resonant wavelengths at each refractive index, while the blue line denotes the linear regression analysis of these data points. Generally, the sensing capability of a sensor is evaluated by its sensitivity (S=Δ λ/Δ n) and FOM (FOM=S/FWHM). In this study, we have calculated the sensitivity S=300 nm/RIU of an ITO nano-resonant ring at the selected parameters, which corresponds to an impressive FOM value of 34.1 1/RIU.

To compare with the dielectric structure, we have also simulated the absorption spectra of ANRR under identical conditions. Similarly, as the refractive index increases, the resonant wavelengths experience redshift, as illustrated in Figure 10c. In Figure 10d, the resonance wavelengths and refractive index functions are plotted to show that the average sensitivity of ANRR is 500 nm/RIU, with a corresponding FOM of only 24 1/RIU. In sensors applications, the FOM is a more meaningful metric used to evaluate sensor quality, and it strongly depends on the resonant bandwidth. The INRR has a narrower FWHM and a greater FOM, indicating the efficient sensing capacity of this structure.

### 3.4. Polarization and Incidence Angle

**Figure 11:**
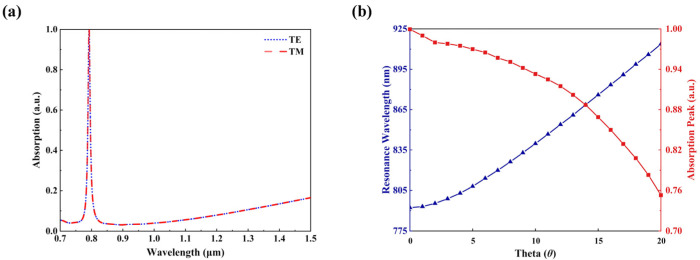
Effects of polarization and incidence angle on absorption characteristics of INRR. (**a**) Absorption spectrum simulation of TE and TM polarization; (**b**) resonant wavelength and absorptivity at different incidence angles.

Finally, we study the effects of variations in the polarization and incidence angle of the refractive sensor. In practical applications, absorption spectra should remain robust when the two parameters are changed [42]. Hence, we investigated the spectral manifestations with respect to different polarization directions and the absorption spectra when varying oblique incidence angles, which are shown in Figure 11a,b. Our findings indicate that there is no discernible difference in absorption characteristics between TM and TE incident light, including when the position of the absorption peak, the absorption rate, and the FWHM remain constant. In nature, this highly polarization-independent property originates from the high degree of geometric symmetry in our designed INRR absorber. Figure 11b exhibits the variations in the resonance wavelength and absorption peak at different incidence angles (0° ≤ θ ≤ 20°). It can be observed that the absorption rates remain above 90% until θ < 13°, indicating the promising practical application of our sensor.

## 4. Conclusions

In summary, this paper has demonstrated a narrowband plasmon nanostructure absorber constructed through the integration of material parameter fitting and simulation approaches, using 3D FDTD to innovatively utilize the INRR of TCO and a Au reflective layer. The results indicate that the absorption structure exhibits complete light absorption at the resonant wavelength of 792.2 nm, with an ultra-narrow bandwidth FWHM of 8.8 nm. By analyzing the distribution characteristics of the electromagnetic field, we find high absorption with a narrow FWHM achieved by the INRR owing to the plasmon resonance exciting the free carries in the surface of the metal and the interior of the ITO. To better show the superiority of TCO materials over traditional metals, we have also designed a pure metal resonant ring ANRR with an identical structure. The comparison between these two structures demonstrates the clear advantages of dielectric materials in such device designs. Additionally, the FDTD simulation investigated the impacts of varying parameters, such as the height h, outer radius R, inner radius r and period P of the INRR, on absorption efficiency. The device exhibits polarization independence owing to its high geometric symmetry, and maintains absorption rates above 90% even at incident angles greater than 13°. As a refractive index detector, it demonstrates a sensitivity of S=300 nm/RIU and a performance coefficient of FOM=34.1 1/RIU, indicating excellent sensing capabilities. This study provides an innovative path for the application of TCOs in a new field, and hopes to provide a clear reference for other related studies in the future.

## Data Availability

Data availability on request from the authors.
